# Remote monitoring of vibrational information in spider webs

**DOI:** 10.1007/s00114-018-1561-1

**Published:** 2018-05-22

**Authors:** B. Mortimer, A. Soler, C. R. Siviour, F. Vollrath

**Affiliations:** 1 Department of Zoology, University of Oxford, Oxford, UK; 2 School of Biological Sciences, University of Bristol, Bristol, UK; 3 Department Continuum Mechanics and Structural Analysis, Universidad Carlos III de Madrid, Madrid, Spain; 4 Department of Engineering Science, University of Oxford, Oxford, UK

**Keywords:** Vibration, Spider, Orb web, FEA model, Vibrometry, Communicated by: Sven Thatje

## Abstract

Spiders are fascinating model species to study information-acquisition strategies, with the web acting as an extension of the animal’s body. Here, we compare the strategies of two orb-weaving spiders that acquire information through vibrations transmitted and filtered in the web. Whereas *Araneus diadematus* monitors web vibration directly on the web, *Zygiella x-notata* uses a signal thread to remotely monitor web vibration from a retreat, which gives added protection. We assess the implications of these two information-acquisition strategies on the quality of vibration information transfer, using laser Doppler vibrometry to measure vibrations of real webs and finite element analysis in computer models of webs. We observed that the signal thread imposed no biologically relevant time penalty for vibration propagation. However, loss of energy (attenuation) was a cost associated with remote monitoring via a signal thread. The findings have implications for the biological use of vibrations by spiders, including the mechanisms to locate and discriminate between vibration sources. We show that orb-weaver spiders are fascinating examples of organisms that modify their physical environment to shape their information-acquisition strategy.

**Electronic supplementary material:**

The online version of this article (10.1007/s00114-018-1561-1) contains supplementary material, which is available to authorized users.

## Introduction

Spider orb webs can be seen as an example of an extended phenotype, i.e. a morphological trait that exists outside and independent of the animal’s body (Blamires 2010; Nakata 2012; Blamires et al. 2017). As such, spider webs are multifunctional structures used by their builders for structural, mechanical and sensory functions (Masters and Markl 1981; Vollrath 1992; Lin et al. 1995; Mortimer et al. 2016).

The sensory functions of the orb web involve propagating vibrations along the converging radial threads, which are used by the spider for information. Biotic sources of vibrations include conspecifics and are used in the context of mating (Maklakov et al. 2003), but also include potential predators (Tarsitano et al. 2000) or prey (Suter 1978; Masters 1984b; Vollrath 1979). Vibrational information can be used to locate and discriminate between these senders and their behaviours to guide behavioural responses. The timing and the amplitude of vibrations can be used in theory to locate where vibrations are coming from, for example by comparing vibration motion between legs (Hergenröder and Barth 1983). In contrast, frequency over time is thought to be used to distinguish between vibration sources, where biotic and abiotic sources show a significant amount of overlap in frequency content (Suter 1978; Masters 1984b; Landolfa and Barth 1996). Spiders will also need to distinguish environmental sources of vibration, such as from wind or moving support structures, which can be used to inform web building choices (Wu and Elias 2014). Importantly for our analysis presented here, a spider’s web must be regarded as an integral part of the animal’s cognitive system (Japyassú and Laland 2017), as the spider can directly influence vibration propagation in the web by changing aspects of silk stiffness, tension and web architecture (Mortimer et al. 2016).

**Fig. 1:**
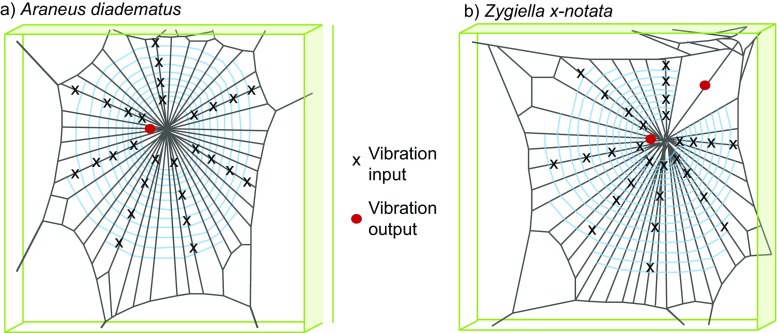
Orb web architecture of **a** *Araneus diadematus* and **b** *Zygiella x-notata*. The x symbols denote vibration input positions. The red circle denotes the vibration output position (laser measurement point) either at the hub (both) or on the signal thread (*Zygiella* only), close to where the spider would usually monitor vibrations

Many orb-weaving spiders reside on the web’s hub (centre) and directly sense web vibrations using sensory input into all eight legs (Fig. 1a); this is a strategy commonly used by the garden cross spider *Araneus diadematus*. Other spiders monitor vibrations from a silken retreat using a signal thread (Fig. 1b)—a strategy evolved several times convergently (Eberhard 1990; Gregoric et al. 2015). The sector web spider *Zygiella x-notata* uses such a signal thread strategy (Mortimer et al. 2015) by having a web sector that has no capture spiral either because of returns in the path or because of threads cut away (Zschokke and Vollrath 1995). Similar to the araneids, *Zygiella* constructs its web daily and it also removes the auxiliary spiral during web construction (Zschokke and Vollrath 1995; Venner et al. 2000).

A signal thread can also be employed by orb weavers lacking a free sector (Foelix 2010). For example, if conditions are unfavourable for monitoring vibrations at the hub, then *Araneus diadematus* may construct a signal thread out of the web plane to connect to a retreat deep in the vegetation (Wirth and Barth 1992). For *Zygiella*, however, the strategy of connecting the web to a sheltering retreat is the rule, which remains hidden during daylight hours, only emerging during prey capture and then rarely staying for long on the hub or web (Klärner and Barth 1982; Pasquet et al. 2007). Mate-guarding males are an exception by hanging around outside the female’s retreat (Bel-Venner and Venner 2006).

A comparison of the webs of garden and sector spiders raises interesting questions regarding the costs and benefits of their respective information-acquisition strategies, which ultimately requires testing in terms of implications for spider survival. Previous studies have shown that building a retreat is a spider’s anti-predator strategy, reducing the risks from a variety of animals, ranging from other spiders to dragonflies, parasitoids and birds (Cloudsley-Thompson 1995). For example, residing in a retreat may reduce the likelihood of parasitoid attacks on *Zygiella* (Pasquet et al. 2007), while also providing good shelter during adverse weather (Heiling 2004).

Still, while a retreat is clearly of benefit to the spider, one might assume that there are also costs associated with being away from the hub and thus the direct contact with all angles of the web. Indeed, it does take *Zygiella* longer than a corresponding hub dweller to respond to prey entrapped and then also to subdue it (Klärner and Barth 1982). This is because the spider has to first move from the retreat to the hub in order to orientate towards the trapped prey, before it can run out to subdue it. Conversely, hub-dwelling spiders position themselves at the hub facing downwards, and this has been modelled to be the best position for successful prey capture, i.e. the optimal place for detecting vibrations in the orb (Maciejewski 2010; Zschokke and Nakata 2010). For the out-of-hub spider, the issue is one of balancing costs and benefits: to initiate movement to the hub, *Zygiella* requires a vibratory stimulus, but misinterpretation should be minimised so that the benefits of hiding outweigh the risks of leaving the retreat.

In this work, we address the question concerning vibrational information-acquisition: how does the on-hub strategy compare versus the off-hub, signal thread strategy? Vibrations radiate from various sources and propagate through the web as transverse, lateral and longitudinal waves, and here, we focus on transverse and longitudinal wave transfer. Vibration displacement of transverse waves is perpendicular to the fibre axis and web frame, while vibration displacement of longitudinal waves is within the axis of the fibre (Masters and Markl 1981). Spider vibration sensors, the slit sensilla, show equal sensitivity for both transverse and longitudinal waves down to c. 10 nm displacement (Liesenfeld 1961; Barth and Geethabali 1982). Longitudinal waves are generated by vibration sources simultaneously with transverse waves (Masters and Markl 1981) and are around an order of magnitude faster than transverse waves through silk (Mortimer et al. 2014), i.e. have shorter propagation times. Wave speeds also differ through the stiffer radial threads and less stiff capture spiral, and waves lose energy over propagation distance and at junctions between threads (Masters 1984a; Landolfa and Barth 1996). Therefore, web architecture alters how vibrational energy spreads through the web structure, where longitudinal waves do not spread as much. Indeed, at the hub, longitudinal waves are known to provide directional information for prey location (Masters and Markl 1981; Masters 1984a; Landolfa and Barth 1996), but it is currently unknown whether the vibration transfer along a signal thread can give clues on prey location. We note that both off-hub and on-hub spiders tend to pluck radial threads on the hub in order to fine-tune the location of prey items (Robinson and Olazarri 1971; Klärner and Barth 1982; Landolfa and Barth 1996).

To measure vibration propagation within the web of the on-hub orb weaver (*Araneus diadematus)* and the off-hub sector web spider (*Zygiella x-notata*), we used a controlled constant vibration source (3 ms pulse from a solenoid). With this, vibrations were applied at specific positions in the different sample webs and laser vibrometry was used to measure the transverse vibration of the web at the positions close to where the spiders would usually reside (Fig. 1). The empirical measurements on transverse and longitudinal waves from our experiments were complemented with finite element analysis (FEA) models of the two web architectures, including models where spider mass is present on the web. This combination of empirical and modelling data allowed us to compare the propagation times, associated wave speeds, as well as the attenuation of vibrations from different areas in the web for these two very different web-monitoring strategies.

## Materials and methods

### Spiders and webs

Spiders of two species, *Araneus diadematus* and *Zygiella x-notata*, were collected during the day over a 2 week period from various urban locations in Oxford City. Spiders were kept in 30 × 30 × 5 cm Perspex frames in lab conditions of c. 20 °C and 40% relative humidity with a 16 h:8 h light–dark cycle. For the key experiments, we used webs built in frames by three different spiders each per species, where all webs were at least the third web or later built under laboratory conditions. Anaesthetised spiders were weighed and photographed on graph paper after their webs were completed. All spiders were handled according to local lab risk assessments/institutional ethical guidelines and do not currently fall under regulation by UK or EU legislation.

The webs were photographed and digitised, and vibration stimulus input positions were then mapped onto the digital webs (Fig. 1a). Distances from the vibration stimulus input positions to the laser position were measured three times using ImageJ software (National Institute of Health). The number of capture spiral junctions per radial thread unit length was also measured across all webs.

### Vibrometry

The vibration stimulus input was provided by a solenoid which produced an approximately square wave loading of 3 ms duration (maximum displacement 0.21 mm), which represents a broadband vibration input as it contains many frequencies simultaneously. Due to the small amplitude and finite duration of the input pulse, as well as high damping within the web, no wave reflections nor resonant peaks are detected (Mortimer et al. 2016), so the propagation of the pulse can be mapped through the web. A metal pin attached to the solenoid (a cylinder of 1 mm diameter) was carefully positioned orthogonally onto the radial threads of the webs using a micromanipulator (Mortimer et al. 2016). The solenoid was moved to different positions on the web, on six to eight different radials, at two to four locations of various distances from the hub (e.g. Fig. 1a). Five measurements were taken per vibration stimulus input position, where data were only included when signal to noise ratios were within a similar range.

Vibrations in the web (without the spider) were measured using laser Doppler vibrometry (Polytec PSV-400). Further details of the vibrometry method are described elsewhere (Mortimer et al. 2016). For transverse wave measurement, the laser was focussed orthogonal to the web. The laser focus point remained stationary—either close to the hub for *Araneus* and *Zygiella* webs or on the signal thread for *Zygiella*, close to where the spiders’ legs naturally sit on a thread (see Fig. 1). A position closer to the retreat was not possible for *Zygiella* without modifying the web due to silk frame threads in the path of the laser. Finally, as a reference, the movement of the solenoid was also measured at each vibration stimulus input position using a PDV-100 vibrometer (Polytec). Both vibrometers were triggered simultaneously from the solenoid voltage input. For some experiments, a mass equivalent to an adult *Araneus* spider (20 mg) was added to the web hub and web vibration was measured with and without the mass for the same web. This was achieved by hooking a weighed mass made from tack onto the hub cross threads.

### Analysis

A custom-written Matlab code was used to analyse all data; the code is described in more detail elsewhere (Mortimer et al. 2016). Local maxima on displacement–time plots were located (i.e. highest amplitude in m that can be compared to the peak amplitude of the vibration input). Damping coefficients were calculated, which is the amplitude loss over distance in decibels per centimetre. Maximum displacement amplitudes for each vibration stimulus input position on a web were converted to attenuation in decibels, where attenuation was relative to the maximum displacement amplitude measured (across any of the webs from either species). The normalised attenuation values were grouped into nine bins of 5 dB (range − 45 to 0 dB) to allow comparison of attenuation within and between webs. In addition, fast Fourier transform (FFT) spectra were calculated for one web, with a frequency resolution of 25 Hz, which were subsequently smoothed using a moving average of 25 points and mean spectra were obtained for different vibration stimulus input positions.

For most displacement–time data, the start of the pulse at the measured position on the web (termed start time) was defined as occurring when five or more consecutive data points over time had magnitudes greater than three standard deviations of that of the pre-triggered data (when no motion was present before the vibration stimulus was applied), where the standard deviation was calculated from 0 to 1.5 ms. The model data from Fig. 4 used a different method to calculate the start time due to low variation in the model output. Here, the physical sensitivity threshold of the spider’s leg was used to determine the start of the pulse, taken as 10 nm for the slit sensilla (Liesenfeld 1961; Barth and Geethabali 1982). The start time was defined as when five or more consecutive data points over time had magnitudes over five times the spiders’ slit sensilla sensitivity. Propagation time was then given as the difference between the mean solenoid start time (input time) and the start time (output time; see Online Resource 1). Transverse wave speed was then calculated by dividing the propagation distance by the propagation time. Standard errors of the mean per vibration stimulus input position (*n* = 5 repeats) were calculated using appropriate propagation of errors. Speeds were not included if the coefficient of variance was over 150% (affecting five vibration stimulus input positions for transverse wave measurements). The density of silk was taken to be 1325 kg m^−3^ (Zemlin 1968). Figures were drawn using OriginPro 8 and Adobe Illustrator CS6 software.

Analyses concentrated on describing the general vibration propagation patterns (i.e. propagation time and attenuation) within individual webs as vibrational stimulus input location changed for the webs from both spider species. The analyses were applied to all three webs of both species, and the conclusions presented applied to webs from all individuals of each species. Differences between spider individuals in terms of silk properties and web architecture (Blamires 2010; Blamires et al. 2017) were expected to have small effects on the vibration propagation patterns in comparison to differences between the species, but are an important aspect deserving further study.

### Modelling

Finite element models were created using the commercial code Abaqus/Explicit 6.14-2. The geometries were modelled from photographs of the same webs of *A. diadematus* and *Z. x-notata* used for vibrometry experiments, where only the hub was simplified.

Anchor threads were pinned at their boundaries, preventing displacements at the ends of the frame strands. The material properties of the silks were purely linear as strains are low (< 1%). Aerodynamic drag forces were introduced following the methodology proposed in Zaera et al. (2014). A pre-tension field was introduced to recreate the tension gradients seen in real webs (Wirth and Barth 1992). Simulations were run with and without additional mass applied. *Araneus* web models used a 20 mg total weight, distributed over eight point masses; the positions of these were obtained from photographs of the spiders in their natural positioning on the real web. *Zygiella* were smaller at 4 mg total weight, and assuming that these spiders place a small amount of their weight on the signal thread, a mass of 0.5 mg was added to the web at the same positions as the measuring point on the experimental data. Data in tables and figures were from experimental measurement unless otherwise indicated. More details on the model and material properties are given in Online Resource 2.

#### Data accessibility

Electronic supplementary material supporting this article is available through download. This includes Online Resources 1–5. Any further data can be requested from the corresponding author.

## Results

### Time and speed

**Fig. 2:**
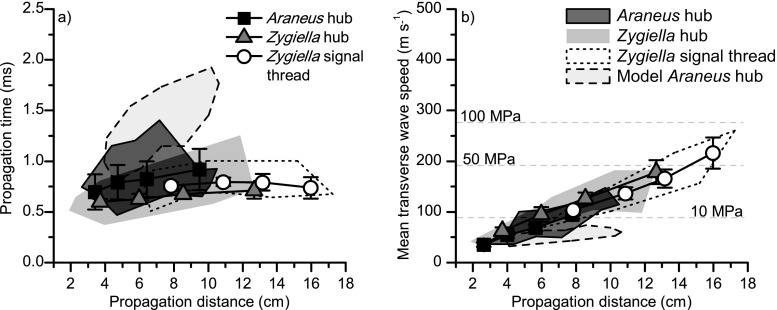
Transverse wave propagation through *Araneus* and *Zygiella* webs without the spider: **a** propagation time versus distance and **b** mean propagation speed versus distance, with corresponding theoretical mean radial thread stress (dashed light-grey lines). Joined scatter points give experimental data from one radial thread for *Araneus* hub, *Zygiella* hub or *Zygiella* signal thread, and error bars give standard error of the mean between repeats for each measured point (*n* = 5). Shaded areas give the envelope encasing data from all vibration stimulus input positions for the *Araneus* hub, *Zygiella* hub, *Zygiella* signal thread and model *Araneus* web at the hub, which had the equivalent geometry and input and output positions to the *Araneus* experimental web

Measurement of web vibration at the hub and at the signal thread for *Araneus* and *Zygiella* webs respectively revealed that the *Zygiella* signal thread had no notable effect on the propagation time (Fig. 2a); neither did the signal thread affect the relationship between wave speed and propagation distance of transverse waves (Fig. 2b). In terms of propagation time, all experimental data points, irrespective of the vibration input or measurement position, had propagation time within a similar range under 1.5 ms (Fig. 2a, Online Resource 1). The output from the models gave a higher range of propagation times under 2 ms. These propagation times thus corresponded to an increased mean propagation speed as the vibration stimulus input position moved further from the hub along a radial thread (Fig. 2b). There was no notable difference between radial threads within a web in terms of propagation time or speed, and similar trends were seen for other webs of both species.

Transverse wave speed can be linked to the mean stress (force per unit area) within the silk threads, as stress is equal to the square of the transverse wave speed, multiplied by silk density (Main 1993; Mortimer et al. 2014). Figure 2b gives corresponding mean stress values (i.e. across the whole propagation path) for a few transverse wave speed values. Importantly, the mean stress values matched the range of tensions directly measured from real webs (Eberhard 1981; Wirth and Barth 1992); this lends support to our method. The reduced wave speeds and so higher propagation times seen in the model webs compared to the equivalent experimental data (Fig. 2) can be explained by lower tensioning of the radial threads in the models, as discussed by Mortimer et al. (2016).

### Attenuation

**Fig. 3:**
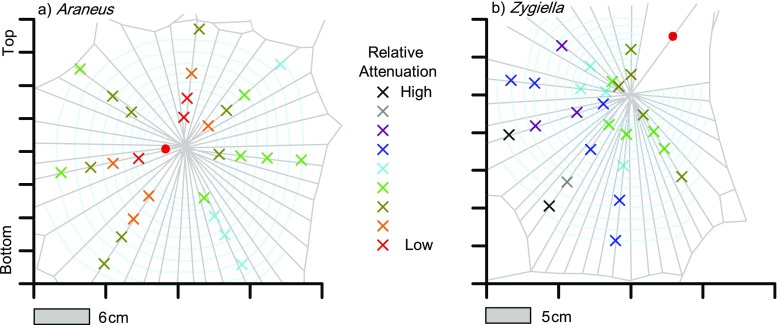
Relative attenuation of transverse waves over different parts of a web of **a** *Araneus* and **b** *Zygiella*. Crosses denote vibration stimulus input positions, and the colour of the cross gives relative attenuation from high (black) to low (red). Each colour represents bins of 5 dB relative to maximum amplitude measured across both webs, from − 45 to 0 dB. Red circles give the vibration output position

The relative attenuation of damped propagating transverse waves at different locations in the capture area for a web of *Araneus* and *Zygiella* is given in Fig. 3. Vibrations measured in *Zygiella* webs at the signal thread were more damped than those measured at the hub in *Araneus* webs (Fig. 3, Online Resource 3). The difference in attenuation between webs was not explained wholly by the additional propagation distance, as attenuation per unit length was lower on average in *Zygiella* webs (Online Resource 3). The attenuation applies evenly to all frequencies under 600 Hz (Online Resource 4), and similar trends can be seen for other webs of both species.

Attenuation along a single radial thread was measurable, as discussed in detail elsewhere (Mortimer et al. 2016). Across all radials where an attenuation gradient could be calculated (*n* = 6 for *Zygiella* and *n* = 13 for *Araneus*), the ranges of attenuations calculated were − 2.25 to − 1.13 dB cm^−1^ and − 3.12 to − 0.79 dB cm^−1^ for *Zygiella* and *Araneus* radial threads respectively. The similar range between species was expected as the number of capture spiral junctions on radial threads was similar, i.e. one junction per 2.04 ± 0.27 mm for *Zygiella* and one per 2.06 ± 0.29 mm for *Araneus* webs.

### Effect of mass on vibration transmission

**Fig. 4:**
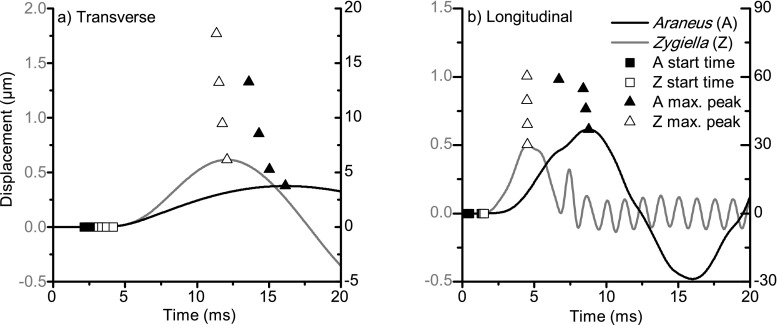
Time–displacement plots for model *Araneus* (black lines and axes) and *Zygiella* (grey lines and axes) webs where spider mass was present on the web: **a** transverse and **b** longitudinal waves. Vibration input was 7.7 cm from the hub for both webs at an angle of 60° relative to the web plane. Vibration output was at the hub for *Araneus*, but on the signal thread for *Zygiella*. Time axis was translated so time = 0 s was the start time of the input. Scatter points give propagation (start) times and maximum peak coordinates of input positions at varying distances from the hub (2.6, 3.9, 5.6 and 7.7 cm)

The presence of the spider on the web would be expected to affect vibration transmission due to the inertia of the spider mass present on the web—mass which is distributed differently in the direct monitoring of *Araneus* versus the remote monitoring of *Zygiella*. Figure 4 shows the time–displacement profiles for transverse and longitudinal waves in *Araneus* and *Zygiella* model webs with spider mass present. Experimental data shown in Online Resource 5 supports the effect of mass on transverse wave propagation.

For both transverse and longitudinal waves, mass affected the propagation times (square scatter points) and the time and magnitude of the maximum peak coordinate (triangle scatter points) and differed between web types (black vs. grey lines; note different axes). For the finite amplitude vibration input used for the model (approx. four times the transverse displacement of struggling prey (Masters 1984b)), the peak amplitudes in *Zygiella* webs would likely be below the threshold sensitivity of this spider (10–100 μm at c. 50–100 Hz (Barth and Geethabali 1982)), further illustrating the attenuation cost of using a signal thread. However, whereas the attenuation of *Zygiella* relative to *Araneus* webs without mass was − 27 and − 36 dB for transverse and longitudinal waves respectively (*n* = 4; identical positions relative to hub), with the mass, the attenuation cost was less for transverse waves at − 16 dB, but was unchanged for longitudinal waves. The time of the maximum peak amplitude for *Araneus* webs was not only slower, i.e. later than for *Zygiella*, but was affected by input position, where a time difference of greater than 2 ms for both transverse and longitudinal waves was seen along a radial (time differences of black triangle scatter points).

## Discussion

Remote monitoring of web vibration used by orb weaver spiders that run a signal thread from the web hub to a silken retreat is an alternative strategy to on-web monitoring (Eberhard 1990; Gregoric et al. 2015). Here, we presented data to measure the principal costs and benefits of using a signal thread for information-acquisition via web vibration. We found that using a signal thread did not increase the propagation time through the web, but it decreased the amplitude of propagating vibrations, and that mass present at the hub affected the timing and amplitude of vibrations. These findings have implications for the biological information that can be gained through each information-acquisition strategy, particularly the vibration source location.

### Cost of a signal thread?

The signal thread off-hub strategy was not accompanied by a time cost of transmission to the spider; the time taken for transverse vibrations to propagate to the spider location either at the hub or on a signal thread was within a similar time range (greatest time difference was 5 ms). The mechanism controlling this is a wave speed gradient within radial threads, which is explained by the presence of the capture spiral, which increases the tension on the radial threads as they diverge (Wirth and Barth 1992; Mortimer et al. 2016). The webs of the two species were similar as they have similar tension gradients, where the signal thread is tensioned by *Zygiella* (Wirth and Barth 1992; Mortimer et al. 2015). The presence of a mass created bigger time differences between the two webs: whereas arrival times were slower for *Zygiella*, as there was no extra tensioning on the *Zygiella* web, the peak times were faster, as there was less of an inertial effect on the *Zygiella* web. But differences between the webs remained under 5 ms, which is small compared to the time needed to behaviourally react to the information. The shortest, i.e. quickest, time that either *Zygiella* or *Araneus* respond to a web vibration has been measured as 100 ms (Klärner and Barth 1982).

Attenuation, or loss of energy, represented an obvious cost of employing a signal thread—vibrations became more damped as they travelled further through silk. This attenuation cost was not as large for transverse waves when mass was present on the webs. Vibrations coming from the capture area opposite the signal thread were attenuated more than vibrations on radial threads closer to the signal thread. This was due to loss of energy as transverse waves propagating through the hub, also evident for *Araneus* webs, where stiff cross threads were present (Zschokke and Vollrath 1995). For *Zygiella*, this suggests that certain radials coupled with the signal thread better, i.e. with less energy loss, than others. Adjustment of radial thread coupling to the signal thread could be one method that *Zygiella* can use to alter the ability of the web to transmit vibrational information.

The signal thread attenuated vibrations in a frequency range under 600 Hz, but it remains to be seen whether this influences the ability of *Zygiella* to discriminate between vibration sources. Frequency-dependent attenuation is likely to change non-linearly for larger-amplitude vibration sources, as damping due to air drag and internal silk damping will increase at higher amplitudes (Kolsky 1964; Sensenig et al. 2012).

Overall, the signal thread attenuates vibrations, particularly those under 600 Hz, but it does not appear to have a cost in terms of the propagation time of vibrations. A spider using a signal thread is therefore likely to require lower amplitude detection thresholds or prey-generated vibrations of higher magnitude to achieve similar prey capture success. This has implications for how the two spiders respond to vibrations, given that time, frequency and amplitude can be used as information to determine the location of the vibration source.

### Locating the vibration source

The location of a vibration source is an important piece of information encoded within web vibration. In theory, time, amplitude or frequency differences between legs or different wave types can be used to determine the vibration source location (Mortimer 2017).

Starting with the timing component, the smallest difference between onset times of two vibrations that the spiders can sense with their slit sensilla has been recorded to be between 2 and 4 ms (Hergenröder and Barth 1983; Barth 1993). We found that the pre-stress gradients in the web resulted in propagation time differences that were so small between different input locations that the spider would not be able to detect any differences. Conversely, if there was no tension gradient along a radial (i.e. a constant low wave speed of 40 m s^−1^), the maximum possible time delay for these web geometries would be 4.4 and 7.2 ms to the hub (17.6 cm) and signal thread (28.8 cm) respectively. The capture spiral is therefore not only important for prey retention (Foelix 2010), but also has significant implications for vibration transmission as it affects pre-stress of the radials (Mortimer et al. 2016).

The fast speeds of both longitudinal and transverse waves would imply that it is highly unlikely that the arrival time can be used for determining the vibration source location, whether at the hub or on the signal thread, as propagation time differences between transverse and longitudinal waves are too short to be determined as separate arrival events (Hergenröder and Barth 1983; Barth 1993). Interestingly, placing a mass at the hub slowed down the arrival time of the peak amplitude of transverse waves sufficiently so that (i) the difference in peak time of longitudinal and transverse waves changed as a function of vibration input location and (ii) the difference in peak time between the locations was greater than the time detection threshold (2 ms). This observation requires further study, as transverse and longitudinal wave peak times may be useful information for prey localisation—when the spider is present at the hub.

Vibration amplitude is likely to be a richer source of information than vibration speed. Longitudinal waves are known to be directional cues (Masters and Markl 1981; Masters 1984a; Landolfa and Barth 1996), but it is unknown whether spiders can determine vibration source distance. For *Zygiella*, both transverse and longitudinal waves are likely to be important for triggering movement out of the retreat onto the hub, where sufficient amplitude is a possible trigger for a predatory response (Liesenfeld 1956; Klärner and Barth 1982; Masters 1984b). We note that *Zygiella* often orientates upon arrival at the hub (Klärner and Barth 1982). However, signal thread vibration may still encode locational information. This is a topic worth further study given the potential applications of vibration-sensing technologies (Tiwana et al. 2012; Fratzl and Barth 2009).

### Conclusions

The observed patterns of vibration transmission highlight how their speed (time differentials) as well as amplitude and frequency could potentially be used by spiders to assess different sources of information in order to maximise prey capture success. The fast propagation speed of both wave types within the web would suggest that spiders are limited by the speed of their physiological processing of vibrational information rather than by the speed of physical propagation of wave information in the web (Klärner and Barth 1982). Longitudinal wave amplitude would provide information for orientation at the hub (Masters and Markl 1981; Masters 1984a), and differences in peak amplitude time and, more likely, amplitude between transverse and longitudinal waves could be used for determining distance to a vibration source. And as Masters (1984b) and Landolfa and Barth (1996) have postulated, spiders should be able to discriminate vibrational signals to inform decision making using both frequency and amplitude. The ability to accurately locate and discriminate vibration sources is likely to have fitness consequences for the spider, and future studies should compare *Araneus* and *Zygiella* in this respect to quantify the selection-relevant fitness costs for *Zygiella* when employing a signal thread.

As has become apparent, many factors determine the costs and benefits of employing the remote information-acquisition strategy of the off-hub spider such as *Zygiella*, where the vibrational information transfer is the key component. Our study highlights how wave propagation is affected by the delicate balance between physical limitations, e.g. attenuation, and factors under the spiders’ control, e.g. web geometry and radial thread tensioning. This shows how the spider’s web is a fascinating example of a biological structure engineered by evolution to enable the spider to balance the many benefits and costs of its extended phenotype.

## Electronic supplementary material

ESM 1 (PDF 586 kb)

ESM 2 (PDF 145 kb)

ESM 3 (PDF 125 kb)
