# Supplementary material for: Remote monitoring of vibrational information in spider webs
Source: Naturwissenschaften. 2018 May 22;105(5):37. doi: 10.1007/s00114-018-1561-1 (PMC5978847; doi:10.1007/s00114-018-1561-1)
Supplement: Supplementary file 1 — (PDF 586 kb) [file 114_2018_1561_MOESM1_ESM.pdf]

Article title: Remote monitoring of vibrational information in spider webs.
Journal name: The Science of Nature.
Author names: B. Mortimer, A. Soler, C. R. Siviour and F. Vollrath.
Corresponding author affiliations: Department of Zoology, University of Oxford, Oxford, UK, School of Biological Sciences, University of Bristol, Bristol, UK.
Corresponding author email: beth.mortimer@zoo.ox.ac.uk.

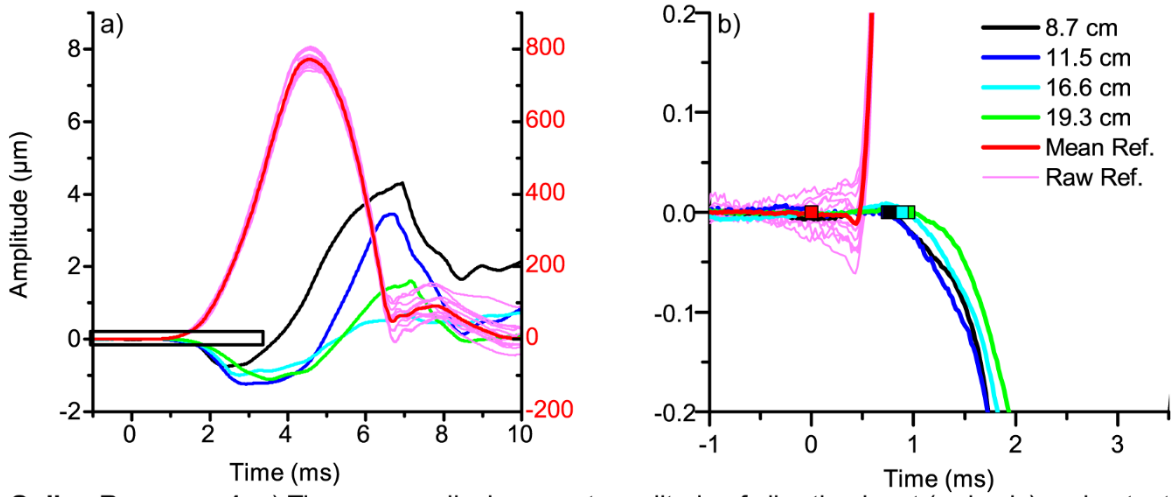

Online Resource 1. a) Time versus displacement amplitude of vibration input (red axis) and output (signal thread, black axis), and b) subset of the same data. In both, vibration input was moved along one radial on a *Zygiella* web. Time axis was translated so time = 0 s was the start time for the input. Scatter points in b give starting time for signal thread data. Method of calculating starting time was identical for all data shown – the time when five or more data points were above three standard deviations of the amplitude of the first 1.5 ms of data.

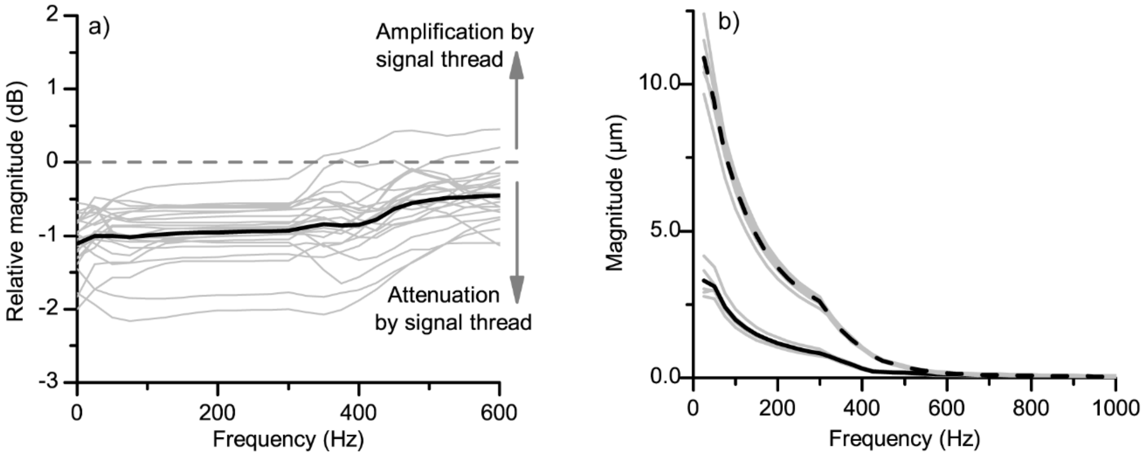

Online Resource 4. Transverse wave frequency filtering within the signal thread of *Zygiella*. a) Each grey line is from a different input location, where the mean curve is given in black. Relative magnitude gives magnitude on the signal thread relative to magnitude at the hub. A value of less than 0 dB (grey dashed line) signifies vibration attenuation due to signal thread use. b) Raw FFT spectra (light grey lines) and mean spectra (black lines) for the same vibration stimulus input position measured at the signal thread (solid line) and hub (dashed line) for a *Zygiella* web.

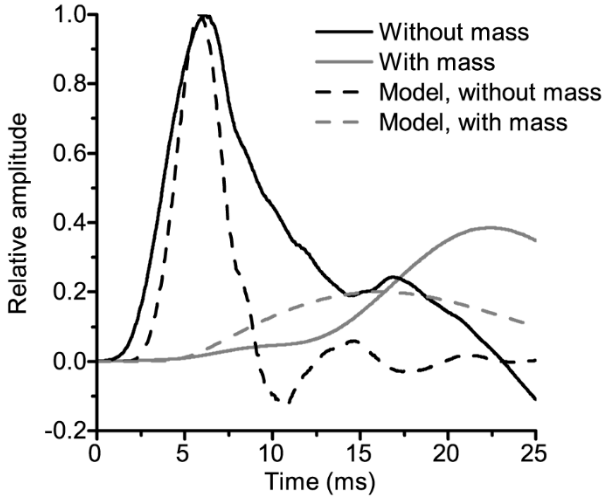

Online Resource 5. Time versus relative displacement amplitude of transverse waves with and without spider mass on *Araneus* webs. Time axis was translated so time = 0 s was the start time of the input. Amplitude is relative to maximum peak amplitude of no mass data.
