# Supplementary material for: Remote monitoring of vibrational information in spider webs
Source: Naturwissenschaften. 2018 May 22;105(5):37. doi: 10.1007/s00114-018-1561-1 (PMC5978847; doi:10.1007/s00114-018-1561-1)
Supplement: Supplementary file 2 — (PDF 145 kb) [file 114_2018_1561_MOESM2_ESM.pdf]

Article title: Remote monitoring of vibrational information in spider webs.
Journal name: The Science of Nature.
Author names: B. Mortimer, A. Soler, C. R. Siviour and F. Vollrath.
Corresponding author affiliations: Department of Zoology, University of Oxford, Oxford, UK, School of Biological Sciences, University of Bristol, Bristol, UK.
Corresponding author email: beth.mortimer@zoo.ox.ac.uk.

Online Resource 2: Supplementary Methods

Finite Element Modelling

The explicit solver of the finite-element code ABAQUS v.6.14-2 was used for the simulations. The short duration of the event, added to the nonlinearities given by the large rotations and aerodynamic drag force make the use of an explicit solver suitable for solving these set of non-linear equations. Silk threads were represented by two-node linear displacement truss elements. The element was defined through a user subroutine, which considers the silk constitutive model, the aerodynamic drag force and the pre-tension field.

In order to model the cable-like behaviour of silk threads, each segment of the web was modelled with at least two elements, introducing an intermediate hinge along each segment, thus capturing the low bending stiffness of such a slender thread. This meshing methodology provides a mechanism that avoids compressive forces, as those induce misalignment of the elements. Furthermore, the accuracy obtained with the proposed mesh have been proven in previous results[1-2].

Silk threads diameters are given in the Supplementary Table below, dimensions varied from *A. diadematus* and *Z. x-notata* considering the relation found between spider mass and thread diameter. Silk mechanical properties were the same for both webs. Spiral threads were implemented with a Young's modulus of 0.06 GPa, while the rest of silk threads were given a Young's modulus of 11 GPa[3-5].

Supplementary Table: Representative diameters for the silks threads

Thread	*A. diadematus* (µm)[5-7]	*Z. x-notata* (µm)[5-7]
Spiral	2.3	1.35
Radii	3.5	2
Frame, mooring, stiff hub and signal threads	5	2.7

The aerodynamic force follows a proper description of the drag force applied to a cylinder, considering the relation between the drag coefficient and the Reynolds number[8-9]. This aerodynamic force is applied as distributed load over the element length, considering thread diameter and velocity (only components orthogonal to the element length).

An iterative algorithm controlling the pre-tension was implemented in the element user subroutine. This method allowed each tread to reach at least the imposed value of stress once the web reached an equilibrated configuration and prior to application of the vibration to the web.

The numerical simulation consisted in two steps. In the first step, as mentioned above, the pre-tension field was applied giving time for the web to reach static equilibrium. This first step was used as a base-state for all the simulations of the same web. Thus, in the second step, the time-displacement excitation recorded in the experimental tests was applied to the web plane, on a node at the same position as the experimental one.

Cited references

1. Zaera R, Soler A, et al. (2014). Uncovering changes in spider orb-web topology owing to aerodynamic effects. J R Soc Interface 11(98): 10. doi 10.1098/rsif.2014.0484
2. Soler A and Zaera R (2016). The secondary frame in spider orb webs: The detail that makes the difference. Scientific Reports 6: 10. doi 10.1038/srep31265
3. Liu Y, Sponner A, et al. (2008). Proline and processing of spider silks. Biomacromolecules 9(1): 116-121. doi 10.1021/bm700877g
4. Gosline JM, Guerette PA, et al. (1999). The mechanical design of spider silks: From fibroin sequence to mechanical function. J Exp Biol 202(23): 3295-3303
5. Köhler T and Vollrath F (1995). Thread biomechanics in the two orb weaving spiders *Araneus diadematus* (Araneae, Araneidae) and *Uloborus walckenaerius* (Araneae, Uloboridae). J Exp Zool 271(1): 1-17. doi 10.1002/jez.1402710102
6. Denny M (1976). The physical properties of spider's silk and their role in design of orb-webs. J Exp Biol 65(2): 483-506
7. Wirth E and Barth FG (1992). Forces in the spider orb web. J Comp Physiol A 171: 359-371. doi 10.1007/BF00223966
8. Tritton DJ (1959). Experiments on the flow past a circular cylinder at low reynolds number. J Fluid Mech 37: 574-567. doi 10.1017/S0022112059000829
9. Jayaweera KOLF and Mason BJ (1965). The behaviour of freely falling cylinders and cones in a viscous fluid. J Fluid Mech 29: 709-720. doi 10.1017/S002211206500109X
