# Supplementary material for: Remote monitoring of vibrational information in spider webs
Source: Naturwissenschaften. 2018 May 22;105(5):37. doi: 10.1007/s00114-018-1561-1 (PMC5978847; doi:10.1007/s00114-018-1561-1)
Supplement: Supplementary file 3 — (PDF 125 kb) [file 114_2018_1561_MOESM3_ESM.pdf]

Article title: Remote monitoring of vibrational information in spider webs.
Journal name: The Science of Nature.
Author names: B. Mortimer, A. Soler, C. R. Siviour and F. Vollrath.
Corresponding author affiliations: Department of Zoology, University of Oxford, Oxford, UK, School of Biological Sciences, University of Bristol, Bristol, UK.
Corresponding author email: beth.mortimer@zoo.ox.ac.uk.

Online Resource 3. Attenuation of transverse wave maximum peak amplitude in *Zygiella* and *Araneus* webs of a fixed vibration stimulus input radius 6.0 ± 0.4 cm from the hub. *Zygiella* web vibration was measured at the signal thread, whereas *Araneus* web vibration was measured at the hub, so had a shorter propagation distance. One *Zygiella* web was measured both at the signal thread and at the hub.

Species	Web	No. measurements c. 6 cm from hub	Attenuation of input c. 6 cm from hub (dB)	Attenuation normalised by distance (dB cm^{-1})	Attenuation due to signal thread (dB)
Zygiella	1	2	-4.49 ± 0.6	-0.38 ± 0.03	
Zygiella	2	3	-4.40 ± 0.6	-0.39 ± 0.04	-1.37 ± 0.91
Zygiella	3	4	-3.78 ± 0.9	-0.33 ± 0.08	
Araneus	1	5	-3.55 ± 0.5	-0.62 ± 0.06	
Araneus	2	5	-3.26 ± 0.3	-0.54 ± 0.05	
Araneus	3	2	-3.08 ± 0.5	-0.50 ± 0.05	
